# Characterization of Variations in PB2, NS1, M, Neuraminidase and Hemagglutinin of Influenza A (H3N2) Viruses in Iran

**DOI:** 10.5812/jjm.9089

**Published:** 2014-03-01

**Authors:** Jila Yavarian, Nazanin Zahra Shafiei Jandaghi, Maryam Naseri, Talat Mokhtari Azad

**Affiliations:** 1 Virology Department, School of Public Health, Tehran University of Medical Sciences

**Keywords:** Influenza A Virus, Pathogenicity, H3N2 Subtype

## Abstract

**Background:**

In the influenza A viruses, neuraminidase (NA), hemagglutinin (HA), PB2, NS1 and M are responsible for the disease pathogenicity. The mechanism of pathogenicity differs among these viruses. Binding of host proteases by the viral NA, sequence of HA in the cleavage and receptor-binding sites, number of oligosaccharide side chains of HA, shortening of NA, and substitutions in PB2, NS1 and M genes, all have been suggested as molecular correlates of pathogenicity of influenza viruses.

**Objectives:**

The goal of this study was to find the alterations in genes, which might be responsible in the virus pathogenesis.

**Materials and Methods:**

Reverse transcription-polymerase chain reaction (RT-PCR) and sequencing of HA, NA, PB2, NS and M genes were performed.

**Results:**

In the receptor binding site of HA, Ile-226, Pro-227, Ser-228, and Asp-190 were found. Arg was in the cleavage site of all viruses and 11-12 N-linked glycosylation sites were found. In NS1, Asp-92 and Ala-149 were detected and Lys-627 was found in PB2 of all viruses in this study. Val-15, Thr-139 and Ala-218 of M1 and Val-28, Leu-54 and His-57 were found in M2 gene. At residue 146 of NA, there was N-linked glycosylation, and Ile-222 was found in the enzyme active site.

**Conclusions:**

The changes found in these five genes, compared to other studies, suggest that viruses studied in this research had the ability to bind to Neu Acα2,6 Gal linkage and had low pathogenicity. It is important to mention that these changes were at the amino acid level and studies need to be performed on animals to investigate the significance of these findings.

## 1. Background

Influenza viruses have segmented genome composed of eight single-stranded negative-sense RNAs, encoding 10-11 proteins. Hemagglutinin (HA), PB2 and NS1 are known as major determinants of the host range restriction and pathogenicity of influenza viruses. Neuraminidase (NA) and M genes also play a role in the pathogenesis. HA and NA are two envelope proteins involved in the virus virulence (1). HA plays a role in binding and uptake of the virus into the cells. It is synthesized as a single polypeptide chain (HA0) which gets cleaved by cellular proteases and is essential for the virus infectivity (2). The sequence of HA cleavage site is important in the pathogenicity, since highly-pathogenic H5 and H7 viruses contain multiple basic amino acids in this site, which are recognized by ubiquitous proteases, furin, and PC6. In contrast, avirulent viruses, except for H7N7 equine influenza viruses (3), contain a single arginine residue at the HA cleavage site and are cleaved in only a few organs (4). Receptor-binding specificity of HA is accountable for the host range restriction of influenza viruses (5). Amino acids of the receptor-binding pocket are important in the receptor specificity (6).

The influenza virulence and host range are also affected by the number of oligosaccharide side chains. HAs typically contain five to 11 glycosylation sites that affect the receptor-binding affinity and/or specificity, antigenicity, virulence, and host range (7-9). These carbohydrates are significant for interaction of HA and NA, where an equilibrium is required between the receptor binding activity and virus release (10-12). NA removes the sialic acid from HA of the newly-synthesized virus particles and helps in release of the virus from the infected cells. To act properly, NA must share the specificity with HA. Amino acid substitutions in and around the active site may be important in the specificity of NA (13-15).

NS1 is an interferon (IFN) antagonist that allows efficient virus replication. NS1 proteins may differ among viruses regarding their ability to counteract the cellular IFN system and thereby affect the viral pathogenicity (16). PB2 protein is a component of the viral replication complex, recognized as an important determinant of virulence and host range restriction (17). M protein is the matrix protein. Mutations in this protein are related to growth, host range, and virulent phenotypes. Capacity of influenza A viruses to replicate in the allantoic sac of the chicken embryo has been attributed to the M gene (18).

## 2. Objectives

The ultimate goal of this study was to determine the variations of viral genes associated with virulence and pathogenesis of some influenza A (H3N2) viruses in Iran.

## 3. Materials and Methods

### 3.1. Virus Detection and Isolation

Throat swab specimens from 432 patients with influenza-like illnesses (fever, cough and sore throat) were referred to the National Influenza Centre at the School of Public Health, Tehran University of Medical Sciences, Tehran, Iran. Virus-positive samples were identified by real-time PCR and inoculated into Madin-Darby canine kidney (MDCK) cells for further virus isolation.

### 3.2. RNA Extraction and RT-PCR

RNAs from positive A (H3N2) samples were extracted using a QIAGEN viral RNA Mini Kit (Cat. No. 52906, Germany) at WHO Influenza Centre, National Institute for Medical Research, London. RT-PCR was performed with a QIAGEN One-Step RT-PCR Kit (Cat. No. 210212, Germany) using primers for HA, M, PB2, NS, and NA genes provided by WHO Influenza Centre, National Institute for Medical Research, London. Primer sequences are available upon request. One-Step RT-PCR was performed with cDNA synthesis (60˚C for 1 minute, 50˚C for 30 minutes and 95˚C for 15 minutes), DNA amplification (94˚C for 30 seconds, 50˚C for 30 seconds and 72˚C for 1 minute) for 40 cycles and final extension at 72˚C for 10 minutes. Desirable products were obtained for all genes except for M gene, which seven samples were positive and seven were negative. Afterwards, hemi-nested RT-PCR was performed on negative samples with Platinum-PFX enzyme; the PCR program was: denaturation at 95˚C for 5 minutes, primer annealing at 55˚C for 30 seconds, extension at 72˚C for 1 minutes in 40 amplification cycles, and a 10 minutes of final extension at 68˚C. PCR product lengths were as follows: PB2: 550 base pair (bp), NS: 850 bp, M: 950 bp; NA and HA in two parts: HA: 850 bp and 900 bp, NA: 700 bp and 750 bp. Purification was performed with GFX PCR DNA and Gel Bond Purification Kits (Illustra, 28-9034-71, UK).

### 3.3. Sequencing

BigDye Terminator V1.1 Cycle Sequencing Kit (Cat. No. 4337449, USA) was used for performing the sequencing reactions with the following program: 25 cycles of 30 seconds denaturation at 96˚C, 15 seconds primer annealing at 50˚C, and 4 minutes extension at 60˚C. After ethanol precipitation, MegaBACE 1000 DNA sequencer was used for resolving the reactions. Wisconsin Sequence Analysis package (GCG), version 10, was used for editing and analyzing the sequences. All the obtained sequences were submitted to the GenBank database under Acc. Nos. FJ664618 to FJ664632 and FJ769856-FJ769915.

## 4. Results and Discussion

In 14 A/H3N2 viruses, changes in HA, NA, M, NS, and PB2 genes were analyzed in a three-year study. Affinity of the viral HA protein for the host sialic acid receptor is a major determinant of host range. In general, human influenza viruses preferentially interact with SAα2, 6Gal, whereas avian viruses mostly bind to SAα2, 3Gal. In H3 subtype viruses, a glutamine at position 226 and a glycine at position 228 within the receptor-binding pocket of HA preferentially accommodate the 2,3-linked sialic acid, whereas a leucine or isoleucine and a serine at these positions confer SAα2,6Gal specificity (6, 19). In this study, isolates had mutations in the receptor-binding site (Ile226, Pro227, Ser228, and Asp190) of HA that bound to the sialic acid in human cells.

Significance of HA cleavability for the pathogenicity of influenza viruses is underscored by the finding that acquisition of a highly-cleavable HA, converts avirulent strains to virulent ones. HA proteins of highly-pathogenic viruses have multiple basic amino acids at the cleavage site, identified by ubiquitous proteases. In contrast, HA proteins of avirulent viruses contain a single arginine residue at the HA cleavage site (as found in this study), cleaved in only a few organs and therefore produce localized infections of respiratory and/or intestinal tract, which are usually asymptomatic or mild (3, 4). HA cleavage efficiency can also be affected by the nature of amino acids downstream of the cleavage site (P1). Horimoto and Kawaoka showed that HA cleavage by subtilisin-like proteases is influenced by the downstream P1 amino acid in the absence of upstream P1 sequence alterations. Substitution of glycine with isoleucine, leucine, valine or proline resulted in reduction of HA cleavage by endogenous endoproteases (20) as all specimens in this study had glycine downstream of P1.

Vigerust et al. showed that glycosylation impacts the disease severity as well as the outcome of infection, as N-linked glycosylation attenuates A/H3N2 influenza viruses (1). Some studies have shown that N-linked glycosylation site at residues 154-156 in the HA1 region of the HA molecule is important in the H5N1 pathogenesis and low pathogenic viruses do not possess N-linked glycosylation in this site; however, there were some controversial results showing that presence or absence of this glycosylation site alone could not account for the differences in pathogenicity (21, 22). Chen et al. declared that absence of a glycosylation site at amino acid 154 in the HA gene of H5N1 influenza virus was important in virulence determination (23). Pattern of N-linked glycosylation site in this study was consistent with the low pathogenicity profile in which there were 11-12 N-linked glycosylation sites and no N-linked glycosylation at residues 154-156. Fujimoto et al. explained the genetic profile of influenza A/H3N2 viruses identified in CSF of patients with encephalopathy, showing a substitution, Thr137Phe, involved in the HA receptor binding site. However, more researches are required to confirm that this mutation can actually influence the pathogenicity (24). This substitution was not found in our study.

Smeenk and Brown showed that replacement of a glycine with tryptophan at amino acid 47 of HA2 subunit results in increased virulence (25), although isolates of the present study did not show this change. Isolates of this study did not have Ser227Asp in the HA1, found in A/Hong Kong/213/03 (H5N1) presumably altering the virulence of human H5N1/97 virus in mice (26, 27). Residues 226, 186, and 196 of HA1 are important in the growth of virus in embryonated eggs. Ala196Thr, Val226Ile and Gly186Val increase the growth of virus in these eggs. In the isolates of the present study Val226Ile was found, but these viruses did not grow in the embryonated eggs. Studies showed that Val226Ile, Thr155 and His156 existing in these isolates, prevent the growth of virus in the eggs.

NA, similar to HA, influences the host range. Studies have shown that the NA gene of influenza A/WSN/33 virus is associated with neurovirulence in mice and growth in MDBK cells. Nucleotide sequence analysis has shown that no conserved glycosylation site is present at the position 146 of NA of WSN virus, playing an important role in the neurovirulence of WSN virus in mice (28-30). Some other studies have shown that low-pathogen H5N1 viruses have a threonine at residue 222 of N2 NA, creating a potential glycosylation site (N-X-T) in the enzyme active site (31, 32). Goto and Kawaoka revealed that lack of this glycosylation site along with presence of a carboxyl-terminal Lys453, was related to the binding of plasminogen by NA, which facilitated and increased HA cleavage and resulted in a wide-range tissue tropism of this virus (33). In the NA gene of the viruses studied in our research, N-linked glycosylation at position 146, tyrosine at residue 453, and isoleucine at residue 222, were present in all cases.

Virulence partially depends on the capacity of a virus to escape or repress the host immune response. The NS1 protein has an important role in this process by neutralizing the cellular IFN response (16). Resistance to the antiviral effects of IFN and TNF-α is attributed to Glu92 of the NS1 protein (34, 35). Viruses expressing NS1 protein with Ala149 could suppress the induction of IFN protein in chicken embryo fibroblasts (CEF), but a virus with Val149 substitution does not have the same effect (36). In the NS1 of Iranian A/H3N2 virus, Asp92 and Ala149 were detected.

PB2 protein is involved in the host range restriction. The replication capacity of virus in mammalian cells depends on the amino acid type, present at position 627 of PB2 (36-38). Lys627 and Glu627 are found in PB2 protein of human and avian influenza isolates, respectively. Steel et al. showed that introduction of an Asp701 in conjunction with Lys627Glu can offset the lack of Lys627 for increasing the transmission rate in mammals. Thus, they showed that PB2 amino acids 627 and 701 are important in mammalian inter-host transmission (17). Collectively, different studies have shown that presence of Glu627Lys in PB2 protein helps avian viruses to grow in humans, and presence of lysine at this position is a significant host range determinant (16). Further studies revealed that lysine, and not glutamic acid, at this position facilitates efficient virus replication at 37˚C (the body temperature of mammals). In addition, specific adaptation of the viral polymerase to the mammalian host facilitates the transmission of influenza viruses raised in different species; thus, one viral feature is changing the virus to a pandemic strain (17). We found lysine at position 627 and aspartic acid at position 701 of PB2.

Capacities of influenza A viruses to replicate in the allantoic sac of chicken embryo have been attributed to the M gene (18). All high-yield phenotypes contained threonine in position 218 instead of alanine or valine, present in the low-yield phenotypes. However, four amino acid replacements in the M2 gene, i.e. Ile28Val, Arg54Phe (Leu), Tyr57His, and Gly89Ser were found in the high-yield phenotypes. In this study, isolates showed characteristics of the low-yield viruses: in the M1 gene, at residues 15, 218 and 139, all isolates had valine, alanine and threonine, respectively; in the M2 gene, Val28, Leu54, His57 and Ser89 were found.

Katz et al. showed that amino acids isoleucine or threonine at residue 222 of NA, and isoleucine or valine at residue 15 of M1 protein, were related to high and low pathogenicity, respectively (32). Meanwhile, a single amino acid replacement of threonine with alanine in position 139 of the M1 gene leads to an increased growth and virulence (35). In the present study, val15 and thr139 in M1 showed association with low pathogenicity, but I222 in NA conferred high pathogenicity. Despite all these progresses, factors behind the pathogenicity of some human virus subtypes have not been completely determined and a variety of factors might contribute to their pathogenicity. Although the relatively-small sample size restricted our power of analysis, according to our findings, different mutations in genes are responsible for the pathogenesis. We conclude that all of these isolates can grow efficiently in human respiratory cells, but they are not virulent enough to cause a systemic infection. We suggest performing more researches in this regard, especially on animal models.
